# Supplementary material for: Polyketide synthase gene clusters in the ericoid mycorrhizal fungus Oidiodendron maius: genome-wide prediction, expression profiling, and functional characterization of knockout mutants
Source: Front Plant Sci. 2026 Sep 16;17:1924430. doi: 10.3389/fpls.2026.1924430 (PMC13623903; doi:10.3389/fpls.2026.1924430)
Supplement: Supplementary file 1 [file SupplementaryFile1.zip › Supplementary Materials/Supplementary Folder 1.zip!/Oidma1_AssemblyScaffolds_Repeatmasked/knownclusterblast/cluster56/jgi.p_Oidma1_138870_mibig_hits.html]

| MIBiG Protein | Description | MIBiG Cluster | MiBiG Product | % ID | % Coverage | BLAST Score | E-value |
| --- | --- | --- | --- | --- | --- | --- | --- |
| AMM63174.1 | AniF | BGC0001371 | Echinocandin\_B\_biosynthetic\_gene\_cluster | 44 | 100.641025641 | 267 | 2.6e-71 |
| AFT91391.1 | HtyE | BGC0000338\_c2 | Echinocandin\_B\_biosynthetic\_gene\_cluster | 45 | 99.0384615385 | 266 | 4.5e-71 |
| EPE34347.1 | Clavaminate\_synthase-like\_protein | BGC0001035 | Pneumocandin\_biosynthetic\_gene\_cluster | 41 | 101.282051282 | 262 | 8.4e-70 |
| AGA37270.1 | 2OG-Fe(II)-oxygenase | BGC0000819 | Paraherquamide\_biosynthetic\_gene\_cluster | 40 | 103.846153846 | 228 | 7.9e-60 |
| EPE34350.1 | Clavaminate\_synthase-like\_protein | BGC0001035 | Pneumocandin\_biosynthetic\_gene\_cluster | 40 | 103.525641026 | 223 | 3.3e-58 |
| AFT91382.1 | EcdK | BGC0000338 | Echinocandin\_B\_biosynthetic\_gene\_cluster | 37 | 102.884615385 | 217 | 1.8e-56 |
| AMM63173.1 | AniK | BGC0001371 | Echinocandin\_B\_biosynthetic\_gene\_cluster | 37 | 102.243589744 | 213 | 2.6e-55 |
| AFU82618.1 | 2-oxoglutarate\_dependent\_oxygenase | BGC0000998 | Guadinomine\_biosynthetic\_gene\_cluster | 30 | 70.5128205128 | 110 | 3.1e-24 |
| Solyc07g043420.2 |  | BGC0001319 | alpha-Tomatine\_biosynthetic\_gene\_cluster. | 25 | 90.7051282051 | 85 | 1.1e-16 |
| PGSC0003DMG400011751 |  | BGC0001320 | alpha-Solanine\_/\_alpha-Chaconine\_biosynthetic\_gene\_cluster. | 24 | 90.7051282051 | 80 | 4.5e-15 |
